# Long-Term Vegan Meditation Improved Human Gut Microbiota

**DOI:** 10.1155/2020/9517897

**Published:** 2020-07-05

**Authors:** Wenrui Jia, Jianhua Zhen, Aijie Liu, Jingyun Yuan, Xiaoli Wu, Pengfei Zhao, Lu Zhao, Xiaolu Li, Qingguo Liu, Guangrui Huang, Anlong Xu

**Affiliations:** ^1^School of Life Science, Beijing University of Chinese Medicine, Beijing 102488, China; ^2^School of Acupuncture-Moxibustion and Tuina, Beijing University of Chinese Medicine, Beijing 102488, China

## Abstract

**Objective:**

Meditation has been widely used for the treatment of a variety of psychological, cardiovascular, and digestive diseases as well as chronic pain. Vegetarian diets can effectively prevent hypertension, metabolic diseases such as diabetes and obesity, and certain cancers. Meditation and vegetarian diets have been recognized as components of a healthy lifestyle and have therefore attracted more people around the world. Meditation can help regulate overall health through the neural-endocrine-immune network. Changes in dietary habits can affect the composition of the intestinal flora, which in turn affects human physiology, metabolism, nutrition, and immune function through the bacteria-intestine-brain axis. Here, we aimed to investigate the effect of long-term meditation and vegan diet on human intestinal flora.

**Materials and Methods:**

The present study used 16S rDNA sequencing technology to detect the differences in intestinal flora between 12 healthy vegan subjects receiving long-term meditation training and 12 healthy omnivorous subjects who never received any meditation training.

**Results:**

The results showed that, compared with the subjects in the omnivorous healthy control group who had never received any meditation training, the intestinal flora structure in the people who followed the long-term vegan meditation practices changed significantly. The intersection set between the results of the LEfSe analysis and the Wilcoxon rank sum test includes 14 bacterial genera. These 14 genera are defined as the dominant genera, and the AUC value was 0.92 in the ROC curve, which demonstrates that the 14 genera can be used as a biomarker to distinguish the two groups. Three beneficial bacteria genera (*Bifidobacterium*, *Roseburia*, and *Subdoligranulum*) were significantly enriched in the meditation group with a threshold of 4, according to the LDAs. The functional prediction of differentially enriched intestinal flora showed that the metabolism of tyrosine, propionate, niacin, and nicotinamide in the intestinal micro-organisms in the meditation group was significantly reduced compared with those in the control group, while the biosynthesis of flavones, flavone alcohols, butosin, and neomycin; flavonoid-mediated oocyte maturation; cytoskeleton protein pathways; and antigen processing and presentation were significantly enhanced.

**Conclusions:**

These results indicate that long-term vegan meditation plays a positive role in improving the body's immunity and adjusting endocrine and metabolic levels, enabling the body to be in a state of good health.

## 1. Introduction

Meditation can be defined as a form of psychological training aimed at improving one's core psychological abilities, such as attention and emotional self-regulation, and meditation has been used for millennia to achieve wisdom [1]. In a broad sense, meditation exercises involve monitoring and regulating attention and emotions, focusing outward on specific physical and sensory stimuli and turning inward toward spiritual experiences and the somatic sensation of physical experience [2]. Mindfulness-based intervention (MBI), a method currently popular in the West, originated from the traditional Buddhist meditation system and has developed into an effective clinical treatment strategy. One hundred forty medical schools and universities in the United States have included MBI in their therapeutic, education, and research programs [3]. Many studies have documented the use of meditation for the treatment of a broad range of mental illnesses, such as neuroses, addictive behaviors, and anxiety and depression, as well as the treatment of cardiac and gastrointestinal diseases and chronic pain; meditation as a therapy has achieved positive results [3–6]. Meditation causes changes in the nerves that regulate the structure and function of the brain regions involved in attention, emotion, and self-awareness [7]. Compared with an equal amount of relaxation training, a 2-week meditation training was found to significantly reduce smoking in addicts by approximately 60%. Brain scans of the resting state showed that the activity in the brain region related to self-control increased in the subjects of a meditation group [8]. Meditation has also been shown to improve immune function, lower blood pressure and cortisol levels, increase telomerase activity, reduce levels of specific markers of inflammation, and exert antiaging effects [9–13]. Mindfulness-based meditation can also alleviate the adverse effects of depressive symptoms on gastroesophageal reflux disease, reduce pain in patients with gastroesophageal reflux disease, and improve their quality of life [14].

The Dietetics and Nutrition Association claims that a vegetarian diet is associated with a reduced risk of ischemic heart disease, hypertension, type 2 diabetes, obesity, and certain cancers [15–17]. Animal products may promote systemic and intestinal inflammation and insulin resistance-dependent metabolic diseases [18]. Numerous studies have shown that a vegetarian diet can prevent many chronic diseases [19–23]. The benefits of a vegetarian diet are mainly related to improving certain metabolic parameters (i.e., oxidation balance, lipid distribution, and glucose homeostasis), the imbalance of which acts as a risk factor for certain chronic diseases that can be directly regulated by the diet [24]. Diet is an important factor affecting the structure of human intestinal flora. Intestinal microbes interact with the host to affect human physiology, metabolism, nutrition, and immune function [25]. Studies have found that the intestinal flora is involved in regulating the cardiac metabolic risk caused by diet, while the bacterial community can affect energy extraction, fat deposition, inflammatory status, and insulin sensitivity [26, 27]. The benefits of a vegetarian diet can be mediated by micro-organisms in the gut [28].

In recent years, intestinal flora has become a popular research subject for various diseases. Diet can change the intestinal flora, and intestinal micro-organisms can positively or adversely regulate the body through the bacteria-intestinal-brain axis of the human body, thereby affecting overall health. Meditation can have a positive effect on the human body and psychology, improve the structure and function of the brain, increase the body's immunity, and alleviate inflammation. Does meditation training affect the body by regulating the intestinal flora and therefore act through the bacteria-gut-brain axis?

The present study used 16S rDNA sequencing technology to detect the differences in intestinal flora between 12 healthy vegan subjects receiving long-term meditation training and 12 healthy omnivorous subjects who never received any meditation training. The role of a long-term vegan meditation practice in regulating the intestinal micro-organisms in humans was therefore examined.

## 2. Materials and Methods

### 2.1. Subject Enrollment and Sample Collection

The 12 subjects in the meditation group were recruited from the Meditation Research Center of Xingtai City, Hebei Province, China (receiving long-term vegan meditation training 30 min per day + vegan diet for more than 3 years), while the 12 subjects in the control group came from the healthy population around the Meditation Research Center (omnivores who had never received any meditation training). The meditation instructions were as follows: sit cross-legged on a comfortable cushion; place your right hand on your left hand, with the thumbs facing each other; and place both hands in front of the navel; the spine should be straight and should not bend in any direction; both shoulders should be balanced with slight adduction; the jaw should be slightly open; the tongue should rest on the roof of the mouth; the eyes should gaze downward at the tip of the nose; and breathing should be uniform and natural. All subjects did not take any special drugs, especially antibiotics, in the 3 months before enrollment. Individuals infected with hepatitis B virus, hepatitis C virus, HIV, or Treponema pallidum; those with documented chronic diseases and abnormal indicators in medical reports; pregnant or lactating women; and subjects participating in other clinical studies were all excluded. This study was approved by the Ethics Committee of the Third Affiliated Hospital of Beijing University of Chinese Medicine and was assigned the ethical batch number BZYSY-2019KYKTPJ-20. All subjects signed informed consent. The study was registered at China Clinical Trial Registry with the registration number ChiCTR1900027754.

According to documented clinical protocol, fecal samples were collected, transported to the laboratory on dry ice within four hours, and stored at −80°C for subsequent processing.

### 2.2. DNA Extraction from 16S rDNA Amplicon

Total DNA extraction was performed according to the instructions of the E.Z.N.A.® soil kit (Omega Biotek, Norcross, GA, USA). The DNA concentration and purity were detected by NanoDrop2000, and the quality of DNA extraction was validated by 1% agarose gel electrophoresis; 338F (5′-ACTCCTACGGGAGGCAGCAG-3′) and 806R (5′-GGACTACHVGGGTWTCTAAT-3′) primers were used for PCR amplification of the V3-V4 variable region using the following amplification procedure: predenaturation at 95°C for 3 minutes, amplification for 27 cycles (denaturation at 95°C for 30 s, annealing at 55°C for 30 s, and extension at 72°C for 30 s), and final extension at 72°C for 10 min (PCR instrument: ABI GeneAmp® 9700). The amplification system utilized 20 *μ*L, including 4 *μ*L of 5^*∗*^ FastPfu buffer, 2 *μ*L of 2.5 mM dNTPs, 0.8 *μ*L of primer (5 *μ*M), 0.4 *μ*L of FastPfu polymerase, and 10 ng of DNA template.

### 2.3. Illumina Sequencing and Bioinformatics Analysis

The PCR products were recovered using a 2% agarose gel, purified by AxyPrep DNA Gel Extraction Kit (Axygen Biosciences, Union City, CA, USA), eluted with Tris-HCl, and detected by 2% agarose electrophoresis. The quantification process was performed using QuantiFluor™ -ST (Promega, USA). The purified amplified fragments were used to construct a library of PE2*∗*300 according to the standard operating procedures of the Illumina MiSeq platform (Illumina, San Diego, USA). Steps for constructing the library were as follows: (1) ligating “*Y*”-shaped adapters, (2) using magnetic beads to screen and remove self-ligating fragments in the adapter, (3) using PCR amplification to enrich the library template, and (4) denaturing with sodium hydroxide to produce single-stranded DNA fragments. Sequencing was performed using Illumina's Miseq PE300 platform (Shanghai Meiji Biomedical Technology Co., Ltd.). The raw data was uploaded to the NCBI database (serial number: SRP^*∗∗∗*^).

The originally acquired sequences were quality controlled with Trimmomatic software and stitched using FLASH software. UPARSE software (version 7.1, http://drive5.com/uparse/) was used to perform OTU clustering on sequences based on 97% similarity, and single sequences and inclusions were removed during the clustering process. RDP classifier (http://rdp.cme.msu.edu/) was used to classify and annotate each sequence and compare them with the Silva database (SSU123) with an alignment threshold of 70%.

### 2.4. Statistical Analysis

Statistical analysis was performed using SPSS software (SPSS v25.0, SPSS Inc., Chicago, Illinois, USA). The normality of the distribution of the variables was tested using the Shapiro–Wilk test. The independent sample *t*-test procedure was used to analyze variables of normal distribution. Nonnormally distributed variables were analyzed using the Kruskal–Wallis test. Enumeration data were tested by chi-square test. *P* < 0.05 was considered a statistically significant difference.

## 3. Results

### 3.1. General Characteristics of the Subjects

The subjects in this study consisted of 12 female individuals who received long-term vegan meditation training (meditation group) and 12 female individuals from the omnivorous population (control group) who had never received any meditation training. The average ages of the subjects in the meditation group and the control group were 39.17 ± 6.658 and 40.5 ± 6.142 years old, respectively. The average body mass index (BMI) of the subjects in the meditation group and the control group was 22.35 ± 2.7903 and 22.41 ± 2.3955, respectively. The detailed comparison results of general data such as gender, age, number of meditation years, dietary pattern, and BMI between the two groups are shown in the Supplement Table 1. There was no significant difference between the two groups in terms of gender, age, BMI, and most of the blood biochemical and blood routine indicators (all indicators were within the normal range). However, there were significant differences in terms of the number of years of meditation, food habits, and bedtime, as well as RBC, hemoglobin (HGB), mean corpuscular volume (MCV), platelet distribution width (PDW), alanine aminotransferase (ALT), glutamyl aminotransferase (GGT), total protein (TP), albumin (ALB), and glucose (GLU) (Supplemental Tables 2 and 3). The results showed that the daily bedtime in the meditation group was significantly earlier than that of the control group (*P*=0.000001), and the RBC, HGB, PDW, ALT, GGT, PT, ALB, and GLU in the meditation group were significantly lower than those in the control group (*P*=0.015, *P*=0.031, *P*=0.041, *P*=0.001, *P*=0, *P*=0.004, *P*=0.031, and *P*=0.002, respectively), and the MCV in the meditation group was significantly higher than that in the control group (*P*=0.027).

### 3.2. Structural Characteristics of Intestinal Micro-Organisms in the Meditation and Control Groups

**Figure 1:**
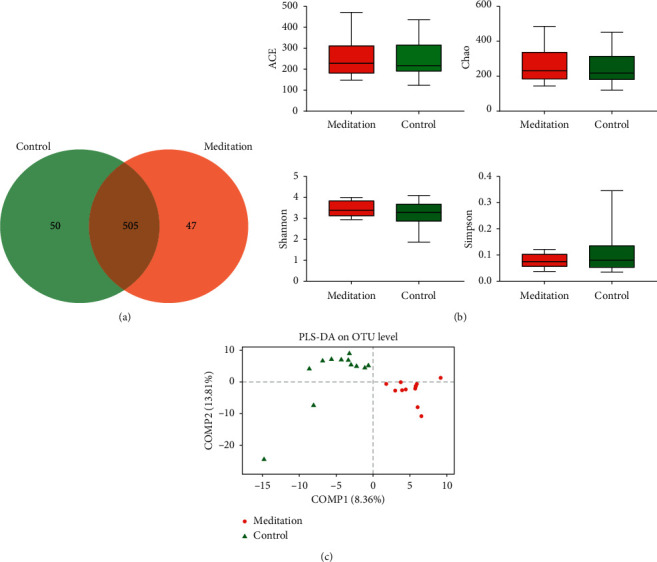
Comparison of intestinal microbial composition and structure between meditation group and control group.

**Figure 2:**
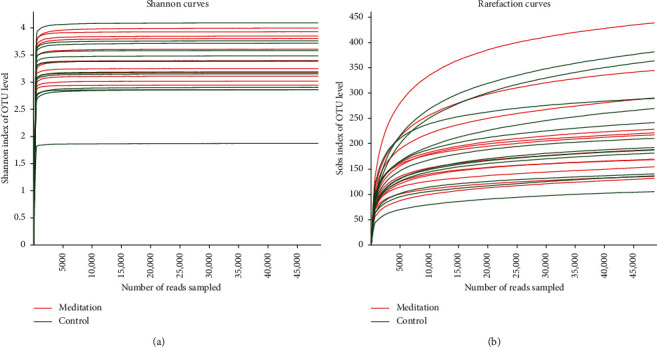
Analysis of intestinal microbial dilution curve in meditation group and control group.

**Figure 3:**
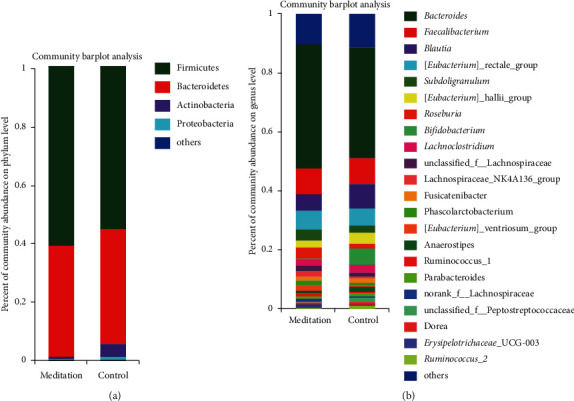
Community composition ratio of intestinal micro-organisms at division and genus levels in subjects of two groups.

Of the 602 OTUs in the two groups, there were a total of 505 OTUs, including 50 unique OTUs in the meditation group and 47 unique OTUs in the control group (Figure 1(a)). There were no significant differences in the ACE and Chao community richness indexes or the Shannon and Simpson community alpha diversity indexes between the two groups (Figure 1(b)). To assess the similarity of the bacterial communities, PCoA and PLS-DA analyses at the OTU levels were performed, which could clearly differentiate the intestinal flora of the long-term meditation group from the control group (Supplemental Figures 1 and 1(c)). The dilution curve analysis based on the sobs index for community richness and the Shannon index for community diversity showed that the sequencing volume had covered all the micro-organisms in the samples and met the data analysis requirements (Figure 2). The community compositions of the intestinal microbes in the meditation group and the control group were analyzed at the division and genus levels. At the division level, the dominant divisions found in both groups were Firmicutes, Bacteroidetes, Actinobacteria, and Proteobacteria (the proportions in the two groups were 60.88%, 37.76%, 0.74%, and 0.56% vs. 55.18%, 38.96%, 4.48%, and 1.36%, resp.) (Figure 3(a)), while at the genus level, *Bacteroides*, *Faecalibacterium*, *Blautia*, [*Eubacterium*] rectale_group, and *Subdoligranulum* were the top five genera (the proportions of which were 42.16%, 8.73%, 5.67%, and 6.50% vs. 37.51%, 8.92%, 8.09%, and 5.92% in the 2 groups, respectively) (Figure 3(b)).

### 3.3. Screening of Different Key Micro-Organisms between the Meditation and Control Groups

**Figure 4:**
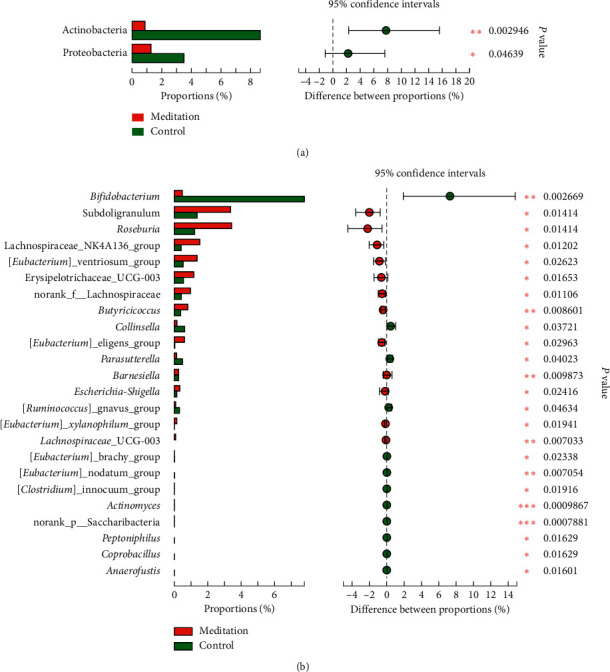
Wilcoxon rank sum test of different genus between two groups. (a) Differential bacteria divisions of intestinal micro-organisms between two groups. (b) Differential bacteria genus of intestinal micro-organisms between two groups.

**Figure 5:**
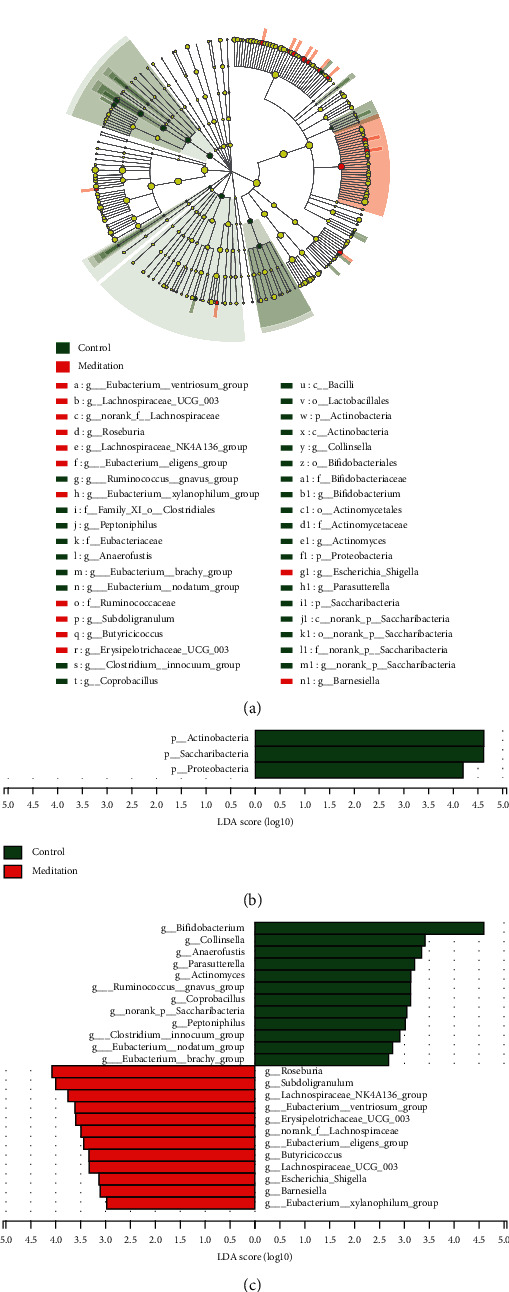
LEfSe analysis to identify the intestinal microbial community at division to genus levels (nonstrict) between the two groups. (a) The Cladogram constructed by the LEfSe method showed phylogenetic distribution of significantly enriched bacteria in the meditation and control groups. Different colored nodes represent microbial class groups that are significantly enriched in the corresponding groups and have significant effects on the differences between groups. B and C LDA bar graphs were used to count the microbial class groups with significant effects in the two groups. LDA scores were obtained through LDA, and the larger the LDA score, the greater the effect of the abundance of representative species on the differential effects. (b) Division level. (c) Genus level.

The Wilcoxon rank sum test of differential species between the 2 groups showed significant changes in the intestinal microbes between the meditation group and the control group (Figures 4(a) and 4(b)). At the division level, the relative percentages of actinobacteria and proteobacteria in the meditation group were significantly lower than those in the control group (*P* < 0.01, *P* < 0.05) (Figure 4(a)). At the genus level, the percentage of *Bifidobacterium* in the meditation group was significantly lower than that in the control group (*P* < 0.01), and the percentages of *Subdoligranulum*, *Roseburia*, Lachnospiraceae_NK4A136_group, [*Eubacterium*] _ventriosum_group, Erysipelotrichaceae_UCG-003, norank_f__Lachnospiraceae, and *Butyricicoccus* were significantly higher than those in the control group (*P* < 0.05, *P* < 0.05, *P* < 0.05, *P* < 0.05, *P* < 0.05, *P* < 0.05, and *P* < 0.01, respectively) (Figure 4(b)). LEfSe analysis identified (threshold 2) the differential intestinal microbial communities between the two groups at the division level down to the genus level (Figures 5(a) and 5(b)). At the division level, Actinobacteria, Saccharibacteria, and Proteobacteria were significantly enriched in the control group (Figure 5(b)). At the genus level, the abundances of 12 taxa in the samples of the meditation group were significantly higher than those in the control group, including *Roseburia*, *Subdoligranulum*, the Lachnospiraceae NK4A136 group, the *Eubacterium ventriosum* group, Erysipelotrichaceae UCG_003, norank_f__Lachnospiraceae, the *Eubacterium*__eligens_group, *Butyricicoccus*, Lachnospiraceae_UCG_003, *Escherichia*_*Shigella*, *Barnesiella*, and the *Eubacterium*__*xylanophilum*_group (Figure 5(c)). The taxa of *Bifidobacterium*, *Collinsella*, _*Anaerofustis*, *Parasutterella*, *Actinomyces*, the *Ruminococcus*__gnavus_group, *Coprobacillus*, norank_p__Saccharibacteria, *Peptoniphilus*, the *Clostridium*__innocuum_group, *Eubacterium*__nodatum_group, and the *Eubacterium*__brachy_group were significantly higher in the meditation group (Figure 5(c)). When the threshold for LDA was increased to 4, the genera enriched at the genus level were *Roseburia* and *Subdoligranulum* in the meditation group and *Bifidobacterium* in the control group (Supplement Figure 2). The intersection set was acquired between the significantly enriched genus identified by LEfSe analysis and those identified by the Wilcoxon rank sum test, and 14 genes were identified as the dominant genus, including g__*Butyricicoccus*, g__*Roseburia*, g__*Bifidobacterium*, g__*Peptoniphilus*, g__*Coprobacillus*, g__nochari_p__Saccharibacteria, g__*Barnesiella*, g__*Anaerofustis*, g__Lachnospiraceae_NK4A136_group, g__*Subdoligranulum*, g__*Actinomyces*, g__*Collinsella*, g__norank_f__Lachnospiraceae, and g__*Parasutterella*. Using the Metagenomeseq differential analysis to compare the differences between the two groups of samples, the significantly different genera (*P* < 0.05) between the two groups were also similar (Supplement Table 4).

### 3.4. Prediction of Intestinal Microbial Function in the Meditation and Control Groups

**Figure 6:**
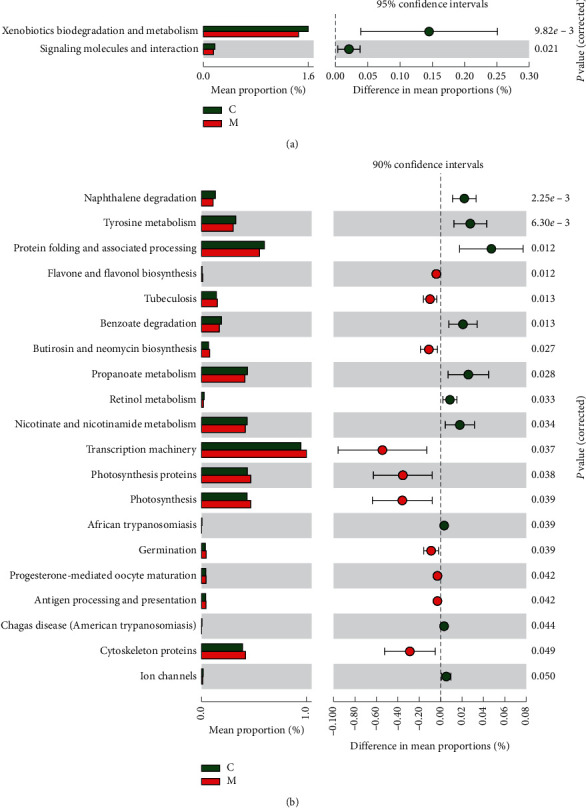
Using PICRUSt to infer and predict the differences in the level 2 and level 3 KEGG functional pathways by 16S rDNA gene sequencing.

Prediction of the genome and function of bacteria are based on 16S rDNA gene sequence. It was found that, at the level of the grade 2 functional pathway, the intestinal microbes in the meditation group showed a significant decrease in the biodegradation and metabolism of exogenous organisms and the enrichment of signal molecules and their interaction pathways (Figure 6(a)). At the level of the grade 3 functional pathway, intestinal micro-organisms in the subjects of the meditation group were observed to have a significant decrease in naphthalene degradation, tyrosine metabolism, protein folding and related processing, benzoic acid degradation, propionic acid metabolism, retinol metabolism, nicotinic acid and nicotinamide metabolism, ion channel, and other metabolism pathways enrichment, but they showed significant enrichment in the biosynthesis of flavones and flavone alcohols, the biosynthesis of butirosin and neomycin, the transcription mechanisms, photosynthetic proteins, photosynthesis, overlap, flavonoid-mediated oocyte maturation, antigen processing and presentation, and cytoskeletal protein pathways. (Figure 6(b)).

### 3.5. Predictive Capability of Intestinal Micro-Organisms in the Meditation and Control Groups

**Figure 7:**
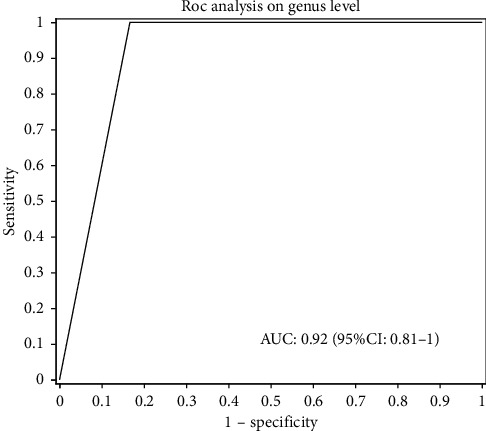
Using the micro-organisms of significant effect in both groups in LDA discrimination and 14 to predict the ROC curve of 14 microbial genera of significant differences in both groups in species differential analysis to predict ROC curve.

To evaluate which microorganismal changes are related to long-term vegan meditation and may be potential biomarkers for meditation subjects, a multivariate regression model based on representative micro-organisms was established, and its identification and prediction capabilities are evaluated using ROC curves. As shown in the figure, a discriminant model based on the representative 14 dominant genera effectively distinguishes the meditation group from the control group (Figure 7). The data of the two models fits well. Therefore, a model based on intestinal microbes can distinguish subjects in the meditation group from subjects in the control group, suggesting that intestinal microbes can predict and identify meditation.

### 3.6. db-RDA Analysis of Intestinal Micro-Organisms in Meditation Group and Control Group

**Figure 8:**
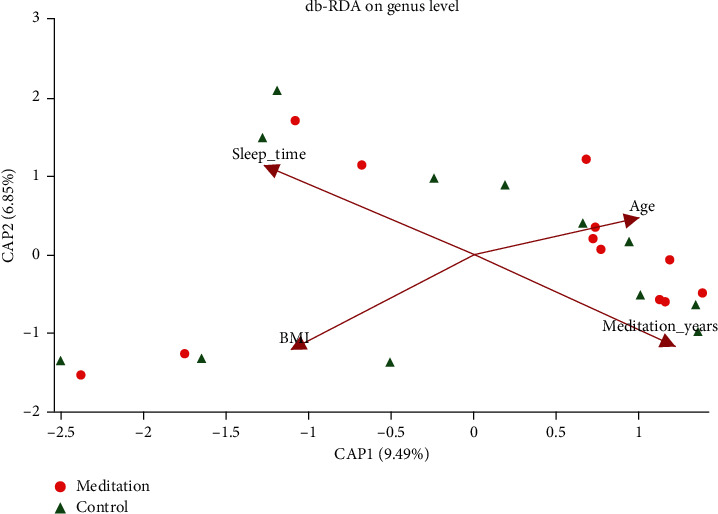
Effects of four environmental factors on the distribution of bacterial communities (person distance algorithm).

According to db-RDA analysis, three environmental factors, such as the number of meditation years, bedtime, and BMI value, have a relatively large impact on the distribution of the sample communities in both groups, and the differences are statistically significant (*P* < 0.05) (Figure 8 and Supplement Table 5).

### 3.7. Relationship between Intestinal Microbes and Clinical Characteristics in Meditation Group and Control Group

**Figure 9:**
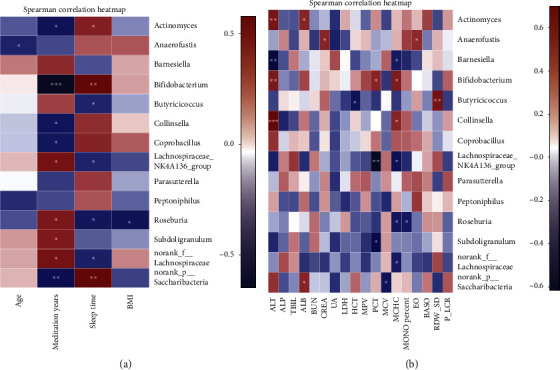
Heat map of correlation between intestinal micro-organisms and clinical characteristics: (a) general characteristics; (b) blood-related indicators.

Correlative assessments were made on the representative 14 dominant bacteria and their environmental factors such as age, meditation years, and bedtime (Figure 9(a)). The results showed that *Anaerofustis* was negatively correlated with age. The numbers of *Actinomyces*, *Bifidobacterium*, *Collinsella*, *Coprobacillus*, and norank_p_Saccharibacteria were negatively related to the number of meditation years. The numbers of Lachnospiraceae_NK4A136_group, *Roseburia*, and *Subdoligranulum*, norank_f_Lachnospiraceae were positively related to the number of meditation years. *Actinomyces*, *Bifidobacterium*, norank_p_Saccharibacteria were positively correlated with bedtime, but *Butyricicoccus*, the Lachnospiraceae_NK4A136_group, *Roseburia*, and norank_f_Lachnospiraceae were negatively correlated with bedtime. *Roseburia* was negatively correlated with the BMI index.

The 38 indicators of routine blood and blood biochemistries were analyzed using the VIF variance expansion factor, and the environmental factors with high autocorrelation were eliminated, and 18 environmental factors with less interaction were retained (Supplement Table 6). Then, the remaining 18 environmental factors were again evaluated for their correlation with the 14 dominant bacteria (Figure 9(b)). The results showed that ALT was positively correlated with *Actinomyces*, *Bifidobacterium*, and *Collinsella* and negatively correlated with *Barnesiella*. ALB was positively correlated with *Actinomyces* and norank_p__Saccharibacteria. CREA was positively correlated with *Anaerofustis*. HCT was negatively correlated with *Butyricicoccus*. PCT was positively correlated with *Bifidobacterium* and negatively correlated with Lachnospiraceae_NK4A136_group and *Subdoligranulum*. MCV was negatively correlated with norank_p__Saccharibacteria. MCHC was positively correlated with *Bifidobacterium* and *Collinsella*, and negatively correlated with *Barnesiella*, the Lachnospiraceae_NK4A136_group, *Roseburia*, and norank_f__Lachnospiraceae. MONO percent was negatively related to *Roseburia*. EO was positively correlated with *Anaerofustis*. RDW_SD was positively correlated with *Butyricicoccus*.

## 4. Discussion

To our knowledge, this is the first study on the regulation of human intestinal flora by meditation. The discriminant model based on the main microflora can effectively distinguish between subjects receiving long-term vegan meditation and healthy omnivores who have never received meditation training. We found that the composition of the intestinal flora in the subjects of the meditation group was significantly different from that of the control group, and the dominant bacterial genera with significant differences between the two groups were significantly related to the number of years of meditation. The results of this study provide new clues for the role of long-term vegan meditation in regulating human intestinal flora.

The fecal microbial diversity estimated by the Shannon and Simpson indexes and the microbial community richness estimated by the ACE and Chao indexes did not show significant differences between the individuals in the meditation group and the control group, which might be due to the subjects all being healthy people; therefore, the alpha diversity of the intestinal microbial communities was not significantly different. However, PCoA and PLS-DA analysis based on the OTU level to evaluate the similarity of the bacterial community can significantly separate the intestinal flora of the long-term meditation group from the control group. Analysis of the composition of the intestinal microbial communities revealed that, at the division level, both groups were dominated by four bacterial divisions, Firmicutes, Bacteroidetes, Actinobacteria, and Proteobacteria. In this study, the relative abundance percentages of Actinobacteria and Proteobacteria in the control group were significantly higher than those in the meditation group, and the relative abundance percentages of Firmicutes were lower than those in the meditation group. Studies have found that the quantity of Proteobacteria in elderly people with Alzheimer's is significantly higher than that of healthy people, and the relative abundance of Firmicutes is significantly lower than that of healthy people [29, 30]. At the genus level, the top five dominant bacterial genera in both groups are *Bacteroides*, *Faecalibacterium*, *Blautia*, [*Eubacterium*] _rectale_group, and *Subdoligranulum*.

When the threshold value was 4, LDA showed that the significantly enriched bacteria in the control group were *Bifidobacterium*, and those in the meditation group were *Roseburia* and *Subdoligranulum*. *Bifidobacterium* is a kind of physiologically beneficial bacteria that has many functions, such as nutrition, enhancing immunity, improving gastrointestinal function, and antiaging. The percentage of *Bifidobacterium* abundance in the meditation group in this study decreased significantly, which is consistent with the results of previous studies on the effect of vegetarian diets on intestinal flora. There are currently three recognized dietary habits around the world: omnivores, ovo-lacto vegetarian, and vegan. An ovo-lacto vegetarian or a vegan diet has a positive impact on human health and can prevent diseases such as cardiovascular disease, cancer, and diabetes, so more people are choosing these dietary habits [31, 32]. Diet also has a significant impact on the composition of the intestinal flora [33]. Many studies have demonstrated that following a vegan diet for more than one year can cause a significant decrease in the number of human intestinal Bifidobacteria [34]. *Roseburia* and *Subdoligranulum* are the significantly enriched genera in the meditation group. Studies have shown that intestinal fibers in vegans and ovo-lacro vegetarians degrade *Roseburia* more significantly than those in omnivores [25]. Studies have also found that both *Subdoligranulum* and *Roseburia* spp. have the ability to ferment in the human intestine to produce short-chain fatty acids, and short-chain fatty acids are generally considered to have a variety of important effects on maintaining human health, such as serving as special nutrition and energy components for the intestinal epithelium, protecting the intestinal mucosal barrier, reducing inflammation, and enhancing gastrointestinal motility. The amounts of these two floras in the meditation group were significantly higher than that in the control group, indicating that long-term vegan meditation can promote the growth of short-chain fatty acid-producing bacteria in the body and play a positive role in health.

The function of the bacterial community was inferred based on 16S rDNA sequencing information. PICUSt and STAMP software were used to analyze the differential functional pathways between the two groups. Compared with the control group, the metabolic functions, such as naphthalene degradation, benzoic acid degradation, tyrosine metabolism, propionic acid metabolism, retinol metabolism, nicotinic acid, and nicotinamide metabolism of intestinal micro-organisms in the meditation group are significantly reduced, while the biosynthesis of flavonoids and flavanols, the biosynthesis of butosin and neomycin, flavonoid-mediated oocyte maturation, the cytoskeleton protein pathway, and antigen processing and presentation are significantly enhanced.

The functional degradation of naphthalene and benzoic acid in the intestinal flora in the meditation group subjects was reduced, which might be due to the reduction of toxins such as naphthalene and benzoic acid in the human body after following the long-term vegan meditation program. The meditation group also demonstrated reduced metabolism of tyrosine, propionate, retinol, niacin and nicotinamide, suggesting that long-term vegan meditation can reduce the body's metabolism and increase its metabolic efficiency. Butirosin and neomycin are aminoglycoside antibiotics, and this class of antibiotics has a broad antibacterial spectrum. Some studies have shown that neomycin has anti-HIV activity, and butirosin exhibits strong antibacterial activity against many Gram-positive bacteria and some Gram-negative bacteria [35, 36]. Some of the micro-organisms in the flora of human bodies can secrete antibiotics under normal conditions, which can inhibit excessive growth of other pathogenic bacteria. Antigen processing and presentation functions belong to the field of acquired immunity, and the biosynthesis of butyricin and neomycin belongs to the field of congenital immunity; the results of this study show that both of the above functions of the intestinal microbes in the meditation group are enhanced, which suggests that meditation can improve the body's immunity. Flavonoids are strong antioxidants that can effectively remove oxygen free radicals in the body, improve blood circulation, and inhibit the exudation of inflammatory biological enzymes [37]. After orally taking or injecting flavonoids, trace amounts of flavonoids in the liver can inhibit the activity of enzymes responsible for drug metabolism to a certain extent [38]. Flavanols have antiallergic, anti-inflammatory, and anticancer properties, and they can increase insulin secretion; many plant-based drugs for treating diabetes are rich in flavanols. Flavanols can also improve immune system function. The biosynthesis of flavonoids and flavanols in the intestinal microbes of the subjects in the meditation group was enhanced, suggesting that meditation may enhance the body's antioxidant, anti-inflammatory, antiallergic, and anticancer capabilities and improve immune system function and circulation. In this study, the levels of blood alanine aminotransferase, glutamyl aminotransferase, and blood glucose levels in the meditation group were significantly lower than those in the control group (*P* < 0.05), but their association with the enhancement of the biosynthesis of flavonoids and flavanols needs to be further explored. Oocyte maturation and fertilization are the core links in animal reproduction, and the cytoskeleton plays a very important role in the normal development and fertilization of mammalian oocytes. The intestinal microbial flavonoid-mediated oocyte maturation and cytoskeletal protein pathway in the meditation group were significantly enhanced, suggesting that long-term vegan meditation may promote oocyte maturation and increase the chance of pregnancy in women.

Prediction models based on 14 dominant bacteria can clearly distinguish between meditation groups and control groups. The results of db-RDA analysis show that the number of meditation years has an important effect on the community structure of the two groups of bacteria (*P*=0.013). Among the 14 dominant genera, Lachnospiraceae_NK4A136_group, *Roseburia*, *Subdoligranulum*, and norank_f_Lachnospiraceae are positively related to the number of meditation years, while *Actinomyces*, *Bifidobacterium*, *Collinsella*, *Coprobacillus*, and norank_p_Saccharibacteria are negatively related to the number of meditation years. *Roseburia* belongs to Firmicutes, which is the main microorganism that produces short-chain fatty acids by fermentation in the human intestine; its production alone can account for 3–15% of the intestinal flora in healthy people [39]. *Roseburia*'s main product is butyric acid. Butyric acid plays an important role in immune regulation and intestinal mucosal barriers. It is the preferred energy source for colon cells and can also maintain the integrity of mucosal membranes and inhibit the inflammatory response by inducing the differentiation of colonic Treg cells [40, 41]. The results of this study show that the blood monocyte ratio is inversely related to *Roseburia* spp., inflammation can cause an increase in the percentage of total monocytes in the body, and *Roseburia* spp. plays an important role in maintaining immunity, which is consistent with previous studies. Other studies have found that the relative abundance of *Roseburia* spp. in the intestinal microbes of mice with acute colitis is significantly lower than that of the mice in the blank control group (*P*=0.0139), and the relative abundance of Lachnospiraceae_NK4A136_group spp. in mice after interventions with prebiotics and probiotics is significantly higher than that in model mice (*P*=0.0344) [42]. Members of the Lachnospiraceae family are involved in maintaining colon health and produce butyric acid to prevent colon cancer in humans [43]. Studies have found that the relative abundance of intestinal flora Lachnospiraceae_NK4A136_group in rats with advanced cognitive decline is significantly lower than that of normal rats with matched age. The results of this study show that Lachnospiraceae_NK4A136_group, *Roseburia*, *Subdoligranulum*, and norank_f_Lachnospiraceae are positively related to the number of years of meditation. It is speculated that long-term vegan meditation can promote the reproduction of these four beneficial bacterial genera in the body, thereby improving the body's immunity.

The putative mechanism for the changes in human intestinal flora caused by meditation might involve the “bacteria-gut-brain” axis, which consists of the central nervous system, autonomic nervous system, enteric nervous system, digestive tract, and intestinal flora. In the “bacteria-gut-brain” axis, signal transductions link the central nervous system and the gastrointestinal tract to form a bidirectional regulation: the bottom-up signal transduction is the afferent fiber projected to the central nervous system and the top-down signal transduction is the projection of the efferent fibers onto the smooth muscle cells of the intestinal wall [44]. When the central nervous system is activated by stress, nerve impulses are transmitted through the downlink signal transduction pathway, which regulate the gastrointestinal motility, digestive enzyme secretion, intestinal mucosal permeability, and the release of signal molecules in the intestinal cavity, and eventually affect the intestinal flora. Murine model experiments have proven that stress, anxiety, depression, and other adverse emotions and mental states can increase intestinal permeability and decrease the body's immunity, which are responsible for the changes in composition and distribution of intestinal flora [45, 46]. In a controlled murine experiment, the researchers compared the intestinal flora of the stress group (rough contact with more aggressive mice) and the control group (kept alone) after 10 days of treatment and found that the biophilia and desalting bacteria of the stress group were significantly higher than that of the control group in the [46]. Stress can also cause physical and chemical changes in the gut, such as changes in stomach acid, mucus, and other intestinal secretions, leading to changes in the environment to which microbiota are accustomed. Anxious mothers had less lactobacillus in their vaginas, an important and beneficial bacterium in the intestines of newborn mice. The mother's inability to pass more lactobacillus to her offspring vaginally during delivery makes them more prone to anxiety and irritability [45]. Therefore, intestinal micro-organisms can not only regulate the nerve system by secreting active substances and directly stimulating the epithelial tissue of the digestive tract, but also change their composition and distribution according to the alterations in intestinal endocrine and dynamics of intestinal mucosal permeability under the downlink signal transduction of the central system.

**Figure 10:**
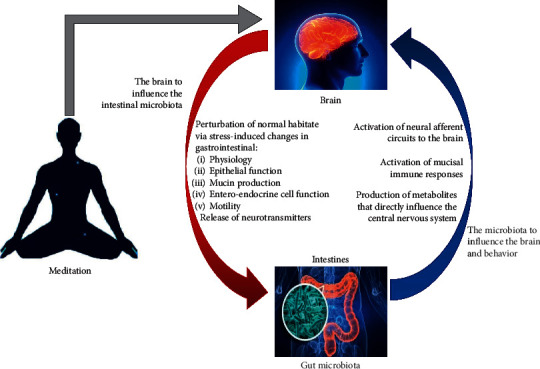
Gut-microbiota-brain bidirectional communication and the possible mechanisms of meditation regulating gut microbiota.

Growing neuroimaging evidence has proven that the practice of meditation causes structural or functional changes in the brain [47, 48]. Meditation training can improve the different functional and structural configurations of the brain's default mode network (DMN), the significance network, and the executive network at rest [8, 49]. Eight-week meditation training can induce overlapping structural and functional effects in the precuneus of the posterior DMN region of the brain, with an increase in cortical thickness and a decrease in low-frequency amplitude (ALFF), while a decrease in ALFF in the left precuneus/posterior cingulate cortex is associated with a decrease in depression scores [50]. Eight-week meditation training is also associated with a significant increase in cortical thickness in the right insula and somatosensory cortex [51]. Meditation training can effectively relieve anxiety, depression, and other adverse mental states [52, 53]. Therefore, we speculated that, by regulating the structure and function of the central nervous system, meditation training may affect the gastrointestinal tract through the downregulation system of the “bacteria-enteric-brain” axis, leading to a series of changes in the intestinal flora (Figure 10).

## 5. Conclusion

The meditation group showed unique fecal-related microbial structure and functional characteristics, suggesting that a long-term vegan meditation practice plays a positive role in improving the body's immunity and adjusting endocrine and metabolic levels. Meditation was therefore found to provide multifaceted and holistic regulation of the body, enabling the body to reach a state of good health. In this study, we considered a strict vegetarian diet and meditation together and defined it as a long-term vegan meditation practice; therefore, its effect on the intestinal flora is caused by the combined effects of diet and meditation, and these two factors may produce mixed effects. In the future, we will study the effects of either factor alone on the intestinal flora and then compare the results with those of the current study to observe the integration of these two interventional factors.

## Data Availability

All data sets generated and analyzed during the whole study are available from the corresponding author on reasonable request.

## Supplementary Materials

Supplementary Materials Supplemental Table 1: general information. Supplemental Table 2: blood routine. Supplemental Table 3: blood biochemical indicators. Supplemental Table 4: MetagenomeSeq Significa. Supplemental Table 5: envfit environmental factor table of person algorithm. Supplemental Table 6: VIF Variance Expansion Factor Analysis Screening Preserves 18 Environmental Factors. Supplemental Figure 1: P CoA analysis of intestinal micro-organisms at OTU level in meditation group and control group. Supplemental Figure 2: LDA discriminant analysis on the significantly enriched genus at genus level at a threshold of 4.
