# Supplementary material for: Long-Term Vegan Meditation Improved Human Gut Microbiota
Source: Evid Based Complement Alternat Med. 2020 Jul 5;2020:9517897. doi: 10.1155/2020/9517897 (PMC7358775; doi:10.1155/2020/9517897)
Supplement: Supplementary Materials — Supplemental Table 1: general information. Supplemental Table 2: blood routine. Supplemental Table 3: blood biochemical indicators. Supplemental Table 4: MetagenomeSeq Significa. Supplemental Table 5: envfit environmental factor table of person algorithm. Supplemental Table 6: VIF Variance Expansion Factor Analysis Screening Preserves 18 Environmental Factors. Supplemental Figure 1: P CoA analysis of intestinal micro-organisms at OTU level in meditation group and control group. Supplemental Figure 2: LDA discriminant analysis on the significantly enriched genus at genus level at a threshold of 4. [file 9517897.f1.zip › 9517897.f1/Supplementary tables.docx]

**Supplementary table 1. General information**

| **Group** | **sex_**  **female**  **(%)** | **omnivore**  **diet(%)** | **vegan**  **diet**  **(%)** | **age(years)** | | | **meditation(years)** | | | **sleep_time(hours/day)** | | | **BMI** | | |
| --- | --- | --- | --- | --- | --- | --- | --- | --- | --- | --- | --- | --- | --- | --- | --- |
|  |  |  |  | **mean** | **SD** | ***P*-value** | **mean** | **SD** | ***P*-value** | **mean** | **SD** | ***P*-value** | **mean** | **SD** | ***P*-value** |
| **Meditation** | 100 | 100% | 0 | 39.17 | 6.658 | 0.615 | 5.83 | 2.368 | 0 | 4.75 | 0.622 | 0.000001 | 22.35 | 2.7903 | 0.957 |
| **Control** | 100 | 0 | 100% | 40.5 | 6.142 |  | 0 | 0 |  | 8.33 | 0.985 |  | 22.41 | 2.3955 |  |

**Supplementary table 5. Envfit environmental factor table of person algorithm**

|  | **CAP1** | **CAP2** | **r2** | ***P*_values** |
| --- | --- | --- | --- | --- |
| **age** | 0.8553 | 0.5182 | 0.1192 | 0.266 |
| **meditation_years** | 0.6029 | -0.7978 | 0.3477 | 0.013 |
| **sleep_time** | -0.6278 | 0.7783 | 0.3509 | 0.01 |
| **BMI** | -0.5354 | -0.8446 | 0.2971 | 0.024 |

**Supplementary table 6. VIF Variance Expansion Factor Analysis Screening Preserves 18 Environmental Factors**

|  | **ALT** | **ALP** | **TBIL** | **ALB** | **BUN** | **CREA** | **UA** | **LDH** | **HCT** | **MPV** | **PCT** | **MCV** | **MCHC** | **MONO%** | **EO%** | **BASO%** | **RDW_SD** | **P_LCR** |
| --- | --- | --- | --- | --- | --- | --- | --- | --- | --- | --- | --- | --- | --- | --- | --- | --- | --- | --- |
| **VIF** | 4.041 | 3.464 | 1.899 | 4.891 | 3.758 | 5.761 | 4.012 | 3.257 | 3.330 | 4.396 | 5.650 | 2.716 | 5.250 | 2.819 | 3.431 | 5.085 | 2.396 | 3.076 |
