# Supplementary material for: Long-Term Vegan Meditation Improved Human Gut Microbiota
Source: Evid Based Complement Alternat Med. 2020 Jul 5;2020:9517897. doi: 10.1155/2020/9517897 (PMC7358775; doi:10.1155/2020/9517897)
Supplement: Supplementary Materials — Supplemental Table 1: general information. Supplemental Table 2: blood routine. Supplemental Table 3: blood biochemical indicators. Supplemental Table 4: MetagenomeSeq Significa. Supplemental Table 5: envfit environmental factor table of person algorithm. Supplemental Table 6: VIF Variance Expansion Factor Analysis Screening Preserves 18 Environmental Factors. Supplemental Figure 1: P CoA analysis of intestinal micro-organisms at OTU level in meditation group and control group. Supplemental Figure 2: LDA discriminant analysis on the significantly enriched genus at genus level at a threshold of 4. [file 9517897.f1.zip › 9517897.f1/supplementary figure.doc]

**Supplementary Figure 1.** PCoA analysis of intestinal microorganisms at OTU level in meditation group and control group

**Supplementary Figure 2.** LDA discriminant analysis on the significantly enriched genus at genus level at a threshold of 4
